# Toxicity and oviposition deterrence of essential oils of *Clinopodium nubigenum* and *Lavandula angustifolia* against the myiasis-inducing blowfly *Lucilia sericata*

**DOI:** 10.1371/journal.pone.0212576

**Published:** 2019-02-20

**Authors:** Stefano Bedini, Guido Flamini, Francesca Cosci, Roberta Ascrizzi, Maria C. Echeverria, Evelin V. Gomez, Lucia Guidi, Marco Landi, Andrea Lucchi, Barbara Conti

**Affiliations:** 1 Department of Agriculture, Food and Environment, University of Pisa, Pisa, Italy; 2 Department of Pharmacy, University of Pisa, Pisa, Italy; 3 Facultad de Ingeniería en Ciencias Agropecuarias y Ambientales, Universidad Técnica del Norte, Ibarra, Ecuador; North Carolina State University, UNITED STATES

## Abstract

Cutaneous myiasis is a severe worldwide medical and veterinary issue. In this trial the essential oil (EO) of the Andean medicinal plant species *Clinopodium nubigenum* (Kunth) Kuntze was evaluated for its bioactivity against the myiasis-inducing blowfly *Lucilia sericata* (Meigen) (Diptera Calliphoridae) and compared with that of the well-known medicinal plant species *Lavandula angustifolia* Mill. The EOs were analysed and tested in laboratory for their oviposition deterrence and toxicity against *L*. *sericata* adults. The physiology of EO toxicity was evaluated by enzymatic inhibition tests. The antibacterial and antifungal properties of the EOs were tested as well. At 0.8 μL cm^-2^, both EOs completely deterred *L*. *sericata* oviposition up to 3 hours. After 24 h, the oviposition deterrence was still 82.7% for *L*. *angustifolia* and the 89.5% for *C*. *nubigenum*. The two EOs were also toxic to eggs and adults of *L*. *sericata*. By contact/fumigation, the EOs, the LC_50_ values against the eggs were 0.07 and 0.48 μL cm^-2^ while, by topical application on the adults, LD_50_ values were 0.278 and 0.393 μL per individual for *C*. *nubigenum* and *L*. *angustifolia* EOs, respectively. Inhibition of acetylcholine esterase of *L*. *sericata* by EOs (IC_50_ = 67.450 and 79.495 mg L^-1^ for *C*. *nubigenum* and *L*. *angustifolia*, respectively) suggested that the neural sites are targets of the EO toxicity. Finally, the observed antibacterial and antifungal properties of *C*. *nubigenum* and *L*. *angustifolia* EOs suggest that they could also help prevent secondary infections.

## Introduction

Myiasis, the parasitic infestation of live mammals by fly larvae (maggots), is an extension of the carrion-feeding habits of blowflies [1]. Gravid females of myiasis-inducing flies such as botfly (Oestridae) and blowfly (Calliphoridae) are attracted and stimulated to lay their eggs on open wounds or even natural body openings of living mammals’ body by a variety of cues, predominantly olfactory ones [2]. On hatching of the eggs, the larvae invade the broken skin and feed on the host’s living or dead tissues and body fluids [1]. Myiasis, is a worldwide severe medical and veterinary problem. In humans, it is a complication of neglected wounds [3; 4]. Particularly in hospitals, the feeding activities of larvae can rapidly lead bedridden patients to develop cutaneous lesions, further oviposition, debilitation, and death. In addition, blowflies can act as carriers of pathogenic bacteria [5; 6; 7]. The larvae of myiasis-inducing flies affect both wild [8] and domestic mammals raising both economic and animal welfare concerns [9]. In animal husbandry across the world, the most common infected host is the domestic sheep, in which cutaneous myiasis or flystrike, is mainly caused by blowflies of the genus *Lucilia* (Diptera Calliphoridae) [10]. Flystrike is a major problem for the sheep industry. It can result in sheep’s serious tissue injuries, loss of productivity and reproductivity and eventually in the animal’s death [11].

In wool-producing countries, flystrike kills millions of heads of sheep a year [12]. In Australia, the annual costs of flystrike, including mortality and loss of production, have been estimated at as high as 280 million A$ [13]. In Great Britain, myiasis was shown to affect 75% of farms [14], with an estimated cost of about 3 million GBP [15] a year.

Currently, the prophylaxis against flystrike relies on synthetic insecticides, such as organophosphates and insect growth regulators (benzoylphenyl ureas, cyromazine and dicyclanil) [16; 17; 12] and, especially for Merino lambs in Australia's extensive wool industry, on painful surgical husbandry procedures such as the docking and the mulesing [18; 19]. However, the side effects of synthetic insecticides, such as the development of insect resistance [20], the harmful effects on sheep [21], farmers [22], and the environment [23], as well as the rising concerns about animal welfare [24] have made alternative strategies a high priority.

**Fig 1:**
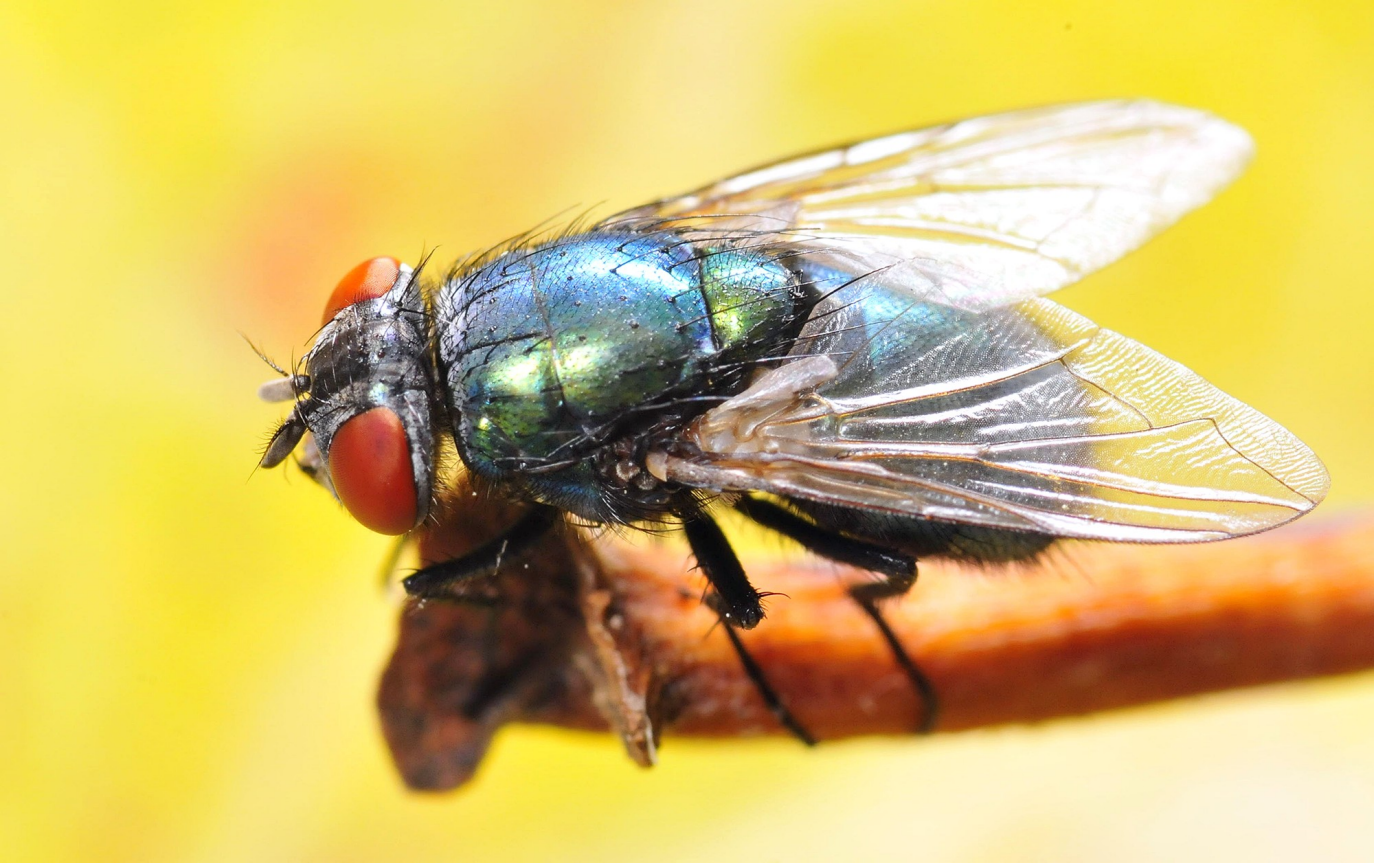
Adult of *Lucilia sericata* (Meigen) (Diptera Calliphoridae).

In recent years, essential oils (EOs) of aromatic plants species have attracted great attention as natural products that can effectively act as insecticides and repellents against insect pests [25; 26; 27; 28; 29]. Moreover, since EOs usually have a low toxicity to mammals [30], and high biodegradability, they are regarded as very promising substances for the formulation of low-toxic, eco-friendly pest control products [31].The common green bottle fly *Lucilia sericata* (Meigen) (Diptera Calliphoridae) (Fig 1) is a common blowfly frequently found in synanthropic and natural ecosystems in most areas of the world and, along with *L*. *cuprina* (Wied.), and *L*. *caesar* (L.), it is a common cause of human and animal cutaneous myiasis [32; 33].

*Clinopodium nubigenum* (Kunth) Kuntze (Lamiaceae) is a typical plant of the high mountains of Ecuador, with an overpowering smell, well known and largely used by local people for its beneficial properties. Such species is widely spread in the Andean region of South America, where it is known as “tipo de cerro” [34]. It is a traditional medical remedy for many communities of the Andean region, for various diseases. As an aqueous infusion, it is used to treat colds and flu symptoms [35; 36], but it is also used to treat digestive disorders and menstrual symptoms [37]. However, its bioactivity is scarcely studied and its effects on insects are still unknown. On the contrary, *Lavandula angustifolia* Mill (Lamiaceae) is one of the main cultivated medicinal plant and its EO is very well-known for its repellent and insecticidal properties against insect pests [38].

In this trial, EOs extracted from *Clinopodium nubigenum* (Kunth) Kuntze and *Lavandula angustifolia* Mill (Lamiaceae) were chemically analyzed and tested in laboratory bioassays for toxicity and oviposition deterrence against *L*. *sericata*. In addition, the physiological mechanisms underlying the toxicity of EOs were investigated by enzymatic inhibition tests as well. Given that blowflies are well-known carriers of harmful microorganisms, the antibacterial and antifungal properties of the EOs against *Bacillus subtilis*, *Candida albicans*, *Escherichia coli*, *Salmonella abaetetuba*, and *Staphylococcus aureus*, which are common pathogens to mammals, including sheep, were analyzed as well.

## Materials and methods

### Essential oils

*C*. *nubigenum* plants were collected with the Ecuadorian Environmental authorization No. 006-2017-IC-FAU-FLO-DPAI/MAE. The essential oil of *C*. *nubigenum* was extracted from the flowering parts of plants collected in the mountains near Hacienda Zuleta (0°12'N, 78°04'W) (Imbaburra, Ecuador) in March 2017 by hydro-distillation in a Clevenger-type apparatus for two hours. The essential oils of *Lavandula angustifolia* was purchased from Sigma Aldrich (Milan, Italy).

### Keeping of the flies

Pupae of *L*. *sericata*, were purchased from Koppert Italia S.R.L. (Verona, Italy), where flies are mass-produced for pollination. The pupae were held in cages until the emergence of the adults which were provided with a solid diet (sugar and yeast 1:1) and water *ad libitum*. The pupae and adults were kept in laboratory conditions (23°C, 60–70% R.H., natural photoperiod).

### GC-MS analysis

The chemical composition of the essential oils of *C*. *nubigenum* and *L*. *angustifolia* was analysed by gas chromatography-electron impact mass spectroscopy (GC-EIMS). The analyses were performed with a Varian CP-3800 gas chromatograph, equipped with a HP-5 capillary column (30 m x 0.25 mm; coating thickness 0.25 μm) and a Varian Saturn 2000 ion trap mass detector. Analytical conditions: injector and transfer line temperatures 220°C and 240°C respectively; oven temperature programmed from 60°C to 240°C at 3°C/min; carrier gas helium at 1 mL/min; injection of 0.2 μL (10% hexane solution); split ratio 1:30. Constituents identification was based on comparison of retention times with those of authentic samples, by comparing their LRIs with the series of *n*-hydrocarbons and using computer matching against commercial (NIST 2014 and Adams 2007) and home-made library mass spectra (built up from pure substances and components of known oils and mass spectra literature data) [39; 40].

### Oviposition deterrence bioassay

One hundred and fifty 10- to 14-day-old unsexed adults of *L*. *sericata* were put in a 75 cm × 75 cm × 115 cm cage (model BugDorm-2400 model of Insect Rearing Tent) with net on the sides and mesh ends at the front. Oviposition was stimulated by polyethylene embedding moulds filled with lamb meat (5 g) mixed with 1 mL of water to prevent desiccation. The meat was gently flattened down and treated with a glass nebulizer with 100 μL of 0, 2, 5, and 10% ethanol solution of the EO, equal to 0.4, 1, and 2 μL cm^-2^. Four groups each of them composed of four meat-moulds treated with the four different EO concentrations (0, 2, 5, and 10%) were put at each corner of the cage about 10 cm from the edge. A beaker containing 500 mL of water, covered by a net, was put in the cage to increase humidity. The eggs laid onto the meat were counted, 3 and 24 h after the start of the test, under a dissection microscope. Large aggregates of eggs were counted by an analytical balance equipped with a piece-counter feature. The test was performed in three replicates. The cages were placed under a bank of fluorescent lamps to provide even lighting and kept at about 23°C and 75% RH.

Oviposition deterrence was calculated using the following formula:

OD%=(NC–NT)/NC*100

Where, OD% = percent oviposition deterrence, *NC* = total number of eggs on the control meat and *NT* = total number of eggs on the treated meat [41; 42].

### Toxicity bioassays

To evaluate the toxicity of the EOs against eggs of *L*. *sericata*, fifty freshly laid eggs (0–12 h old) were placed in a Petri dish (5 cm Ø) the lower surface of which was covered with a black filter paper (Hahnemüehle black filter paper, grade 551) treated with 0.5%, 1.0%, 2.0%, 5.0%, 10.0%, and 20.0% EtOH solutions of the EOs equal to 0.04, 0.08, 0.16, 0.40, 0.80, and 1.60 μL cm^-2^. As control, 50 eggs were placed on filter paper treated with 100 μL of EtOH only. In all of the treatments, before placing the eggs, the ethanol was evaporated by exposing the treated paper to an airflow for 3–5 min and, then, the paper was wetted with 0.4 mL of water. The tests were performed at room temperature (about 23°C). Egg-hatching was recorded after 48 h. Four replicates per concentration were performed.

To evaluate toxicity against adults of *L*. *sericata*, twenty unsexed flies were treated by topical applications of the two EOs. 2 μL ethanol EO solution was applied onto the thorax of each fly with a hand micro-applicator (Burkard Scientific Ltd, Uxbridge, UK) [43]. The EOs were tested at the doses of 0.1, 0.2, 0.4, and 1 μL of EO per insect. Three replicates per dose (60 treated flies) were run. The control flies were treated with 2 μL of ethanol. The treated insects were kept in small Plexiglas cages, 20 cm diameter, 30 cm long (10 insects per cage), with water and sugar *ad libitum* under laboratory conditions (23°C, 75% RH). The mortality of the flies was checked daily for 4 days and values were corrected using the Abbott’s formula [44].

### Acetylcholine esterase inhibition assay

The Acetylcholine esterase (AChE) of *L*. *sericata* was extracted as described by Seo et al. [45] with few modifications. An aliquot (300 mg) of adult insects were homogenized in 4 mL of buffer (10 mM Tris-HCl, pH 8.0) containing 0.5% (v/v) Triton X-100 and 20 mM NaCl. The homogenate was centrifuged at 17,000 g at 4°C for 15 min and the supernatant containing AChE was filtered through glass wool to remove any excess lipid. The total protein content was quantified by the Protein Assay Kit II (Bio-Rad) and the extracted AChE was used in the AChE assays.

Inhibition of AChE was determined by the colorimetric method of Ellman et al. [46] with few modifications using acetylthiocholine (ACh) as substrate. The protein content of the AChE extract was diluted to 0.1 mg mL^-1^ and the reaction mixture consisted of 500 μL of diluted AChE extract (which contained 0.05 mg protein mL^-1^) and 50 μL of EOs for each concentration (2, 5, 25, 50, 100, 125, and 250 mg L^-1^ dissolved in 5% (v/v) acetone). Controls were prepared by adding acetone at the same concentrations and without EOs. The tube was set in incubator at 25°C for 5 min before adding 100 μL of 0.01 M 5,5’-dithiobis-(2-nitrobenzoic acid) (DTNB; dissolved in phosphate buffer pH 7.0) and 2.4 mL of phosphate buffer (pH 8.0). The mixture was gently stirred and incubated for another 10 min at 25°C before adding 40 μL of 75 mM ACh (dissolved in 0.1 M phosphate buffer pH 8.0), then the mixture was incubated for 20 min at 25°C. The activity of AChE was measured by spectrophotometry using an Ultrospec 2100 Pro spectrophotometer (GE Healthcare Ltd, England) at 25°C by the increase of absorbance at 412 nm. Inhibition percentage of AChE activity was calculated as follows:

AChEinhibition%=(1−SAT/SAC)×100

where SAT is the specific activity of the enzyme in the treatment group and SAC is the specific activity of the enzyme in the control group. Residual percentage of AChE activity was calculated as (SAT/SAC) x 100. Three replicates were measured for each EOs concentration.

### Antimicrobial activity assay

The EOs were individually tested against *Escherichia coli* ATCC 10536, *Staphylococcus aureus* (ATCC BAA-1026), *Bacillus subtilis* (ATCC 11774), *Salmonella enterica* subsp. *enterica* serovar Abaetetuba (ATCC 35640), and *Candida albicans* (ATCC 10231). All the strains were purchased from the American Type of Culture Collection (ATCC, Manassan, USA) and maintained in the Laboratories of the Universidad Técnica del Norte, Ecuador. *E*. *coli*, *S*. *aureus*, and *B*. *subtilis* strains were grown on nutrient agar; *C*. *albicans* strain was grown on malt agar; *S*. *enterica* was grown on trypticase soy agar.

The antibacterial activity of the EOs was determined by the agar disc diffusion method as follows: active microbial suspensions were made from 24-h-old agar plates using sterile saline solution up to a concentration of approximately 1–2 x 10^7^ CFU mL^-1^. The microbial suspension was spread on the surface of Mueller Hinton agar (MHA, Oxoid) plates using a sterile cotton swab in order to have uniform microbial growth. Under aseptic conditions, filter paper discs (diameter 6 mm, Whatman paper No.1, Oxoid) were put on the agar plates (one disc per Petri dish, in order to avoid any possible additional activity), then 10 μL of each EO dilutions (corresponding to 10, 5, 2.5, 1.25, and 0.63 μL EOs per disc) were put on the discs. The control discs contained 10 μL of methanol. The inoculated plates were then incubated at 37°C for 24 h to allow microbial growth. Microbial inhibition zones were measured using a digital calliper and given in millimetres (mm). Six repetitions of each treatment were made.

The minimum inhibitory (MIC) and lethal (MLC) concentrations were determined by the broth dilution method in test tubes as follows: 5 mL of 10^7^ UFC mL^-1^ microbial broth was incubated in tubes containing 50 μL of decreasing concentrations of the oil (10, 5, 2.5, 1.25 and, 0.63 μL EOs per tube). The MIC was estimated as the lowest EOs concentration that inhibited any visible microbial growth [47]. To determine the MLC, 0.1 ml of the cell suspensions from the tubes showing no growth were sub-cultured on nutrient agar plates for bacteria and on malt agar plates for yeast to find out if such inhibition was reversible or permanent. The MLC was calculated as the highest EO dilution (lowest concentration) at which no growth occurred on the plates. Three repetitions of each treatment were made.

### Statistics and data analysis

The median lethal dose (LD_50_) of the EOs against *L*. *sericata* adults was calculated by Log-probit regression. Significant differences between the LD_50_ values of the two EOs were determined by estimating the confidence intervals of relative median potency (RMP). The differences were considered statistically significant when values in the 95% confidence interval of relative median potency analyses were ≠ 1.0. The percentages of oviposition deterrence, ovicidal activity, and residual AChE activity were arcsine transformed prior to statistical analysis and processed using GLM with one factor (EO) and dose as covariate. *P* < 0.05 was used for the significance of differences between means. The IC_50_ values of AChE activity (the inhibitory concentration needed to inhibit 50% of the enzyme activity, negative Hill slope) were calculated by nonlinear regression to a four-parameters logistic equation (variable Hill slope). The differences in the sizes of the inhibitory zones formed by the EOs against different microbial strains were tested by the Kruskal-Wallis test and the means were separated by Dunn-Bonferroni pairwise comparisons. Data were processed by SPSS 22.0 software (IBM SPSS Statistics, Armonk, North Castle, New York, USA) and by GraphPad Prism 5 (GraphPad Software, San Diego, CA, USA). The individual data points behind means and variance measurements for the quantitative analyses presented in the tables are available in S1 Appendix.

## Results

### Chemical composition of the EOs

The GC-MS analysis of the EO of *C*. *nubigenum* identified 33 constituents accounting for 99.6% of the whole oil. In the EO of *L*. *angustifolia* 27 constituents were identified, accounting for 99.2% of the whole oil (Table 1). The principal chemical constituent of the EO of *C*. *nubigenum* was carvacrol (32.9%), followed by pulegone (25.4%), whereas linalool (35.2%) and linalyl acetate (33.4%) were the main compounds in the EO of *L*. *angustifolia*. Other important volatiles were p-cymene (9.1%) and iso-menthone (6.4%) for the EO of *C*. *nubigenum*, and α-pinene and borneol (3.6 and 3.5%, respectively) for the EO of *L*. *angustifolia* (Table 1).

**Table 1:** Chemical composition (%) of the *Clinopodium nubigenum* and *Lavandula angustifolia* essential oils used in the assays.

Constituent^a^	*LRI*	*C*. *nubigenum*	*L*. *angustifolia*
tricyclene	928	nd	0.1
α-thujene	931	0.9	nd
α-pinene	941	0.5	3.6
camphene	954	tr	1.3
sabinene	976	0.4	0.4
β-pinene	982	0.5	1.3
3-octanone	988	nd	0.2
myrcene	993	0.3	2.0
3-octanol	993	0.8	nd
α-phellandrene	1005	0.2	nd
δ-3-carene	1011	tr	1.5
α-terpinene	1018	1.0	nd
*p*-cymene	1027	9.1	2.0
limonene	1032	1.5	0.3
1,8-cineole	1034	tr	1.6
(*E*)-β-ocimene	1052	tr	0.1
γ-terpinene	1062	5.3	nd
*cis*-sabinene hydrate	1070	0.6	nd
*trans*-sabinene hydrate	1095	0.1	nd
linalool	1101	0.1	35.2
nonanal	1102	0.1	nd
1-octen-3-yl acetate	1111	0.9	nd
3-octanol acetate	1124	1.0	nd
camphor	1145	nd	0.9
*iso*pulegol	1146	tr	0.2
citronellal	1155	1.8	nd
*iso*menthone	1164	6.4	nd
borneol	1168	nd	3.5
*iso*pulegone	1177	3.5	nd
4-terpineol	1178	tr	2.8
α-terpineol	1191	0.2	0.7
citronellol	1230	1.3	nd
pulegone	1237	25.4	nd
hexyl isovalerate	1244	nd	0.1
piperitone	1252	0.9	nd
linalyl acetate	1259	nd	33.4
lavandulyl acetate	1290	nd	1.3
carvacrol	1298	32.9	nd
eugenol	1358	0.8	nd
piperitone oxide	1363	0.1	nd
neryl acetate	1365	nd	2.6
α-copaene	1376	0.3	nd
citronellyl acetate	1380	0.6	nd
geranyl acetate	1383	nd	0.3
β-caryophyllene	1419	nd	3.3
α-humulene	1455	nd	0.2
(*E*)-β-farnesene	1458	nd	0.5
germacrene D	1481	0.2	0.2
bicyclogermacrene	1495	0.9	nd
lavandulyl isovalerate	1511	nd	0.1
*trans*-γ-cadinene	1514	nd	0.1
δ-cadinene	1524	0.5	nd
spathulenol	1576	0.5	nd
Monoterpene hydrocarbons		19.7	12.2
Oxygenated monoterpenes		74.0	82.4
Sesquiterpene hydrocarbons		1.8	4.3
Oxygenated sesquiterpenes		0.5	nd
Phenylpropanoids		0.8	nd
Other non-terpene derivatives		2.8	0.3
Total identified		99.6	99.2

^a^Chemical constituents ≥ 0.1%; LRI, linear retention index on DB-5 column; nd, not detected; tr, traces.

Monoterpenes, in both their oxygenated and hydrocarbon forms (74 and 19.7%, respectively), represented the main chemical class for the EO of *C*. *nubigenum*. Monoterpenes were also the most abundant chemical class of compounds in the EO of *L*. *angustifolia*, as they accounted for up to 94.6% of the total composition, mostly in their oxygenated form (82.4%) (Table 1).

### Oviposition deterrence

The EOs of *C*. *nubigenum* and *L*. *angustifolia* managed to deter oviposition by *L*. *sericata*. After 3 h, both EOs completely inhibited oviposition (OD% = 100), starting from a dose of 0.4 and 0.8 μL cm^-2^ for *L*. *angustifolia* and *C*. *nubigenum*, respectively, with no differences between the two EOs (*F*_1,16_ = 0.106; *P* = 0.749). After 24 h the most effective EO (*F*_1,16_ = 5.522; *P* = 0.032) was the *C*. *nubigenum* one with an OD% ranging from 72.6 to 89.5, while the OD% of the *L*. *angustifolia* ranged from 7.2 to 82.7, at 0.2 and 0.8 μL cm^-2^, respectively (Table 2).

**Table 2:** Oviposition deterrent effect of *Clinopodium nubigenum* and *Lavandula angustifolia* essential oils (EOs) against *Lucilia sericata*.

EO	Dose^a^	Time^b^	No. of eggs	OD%
*C*. *nubigenum*	0.0	3	675.67 ± 62.65	0.00 ± 0.00
	0.2	3	23.00 ± 14.22	98.84 ± 0.67
	0.4	3	1.67 ± 1.67	99.90 ± 0.10
	0.8	3	0.00 ± 0.00	100.00 ± 0.00
	0.0	24	2169.33 ± 266.21	0.00 ± 0.00
	0.2	24	589.33 ± 224.38	72.61 ± 10.69
	0.4	24	280.44 ± 208.65	84.58 ± 12.29
	0.8	24	183.78 ± 183.78	89.47 ± 10.53
*L*. *angustifolia*	0.0	3	318.67 ± 138.70	0.00 ± 0.00
	0.2	3	95.67 ± 95.67	92.89 ± 7.11
	0.4	3	0.00 ± 0.00	100.00 ± 0.00
	0.8	3	0.00 ± 0.00	100.00 ± 0.00
	0.0	24	1386.56 ± 421.49	0.00 ± 0.00
	0.2	24	1308.56 ± 434.38	7.22 ± 2.84
	0.4	24	235.00 ± 147.56	66.10 ± 8.46
	0.8	24	516.11 ± 271.50	82.70 ± 10.88

^a^, μL cm^-2^

^b^, time after the treatment (h). Data are given as means ± standard error. OD%, percent oviposition deterrence.

### Ovicidal activity

The EOs of *C*. *nubigenum* and *L*. *angustifolia* were found to have a definitely toxic effect on the eggs of *L*. *sericata*. The ovicidal activity was dependent on the EOs (*F*_1,45_ = 38.354; *P* < 0.001) and on the dose (*F*_1,45_ = 74.261; *P* < 0.001). The most effective EO was the *C*. *nubigenum* one with an LC_50_ value of 0.07 μL cm^-2^ while the LC_50_ value of the EO of *L*. *angustifolia* was 0.48 μL cm^-2^ (Table 3).

**Table 3:** Toxicity of *Clinopodium nubigenum* and *Lavandula angustifolia* essential oils (EOs) to eggs of *Lucilia sericata*.

EO	LC_50_^a^	95% CI	Slope ± SE	Intercept ± SE	*χ*2 (df)	*P*
*C*. *nubigenum*	0.07	0.01–0.16	1.87 ± 0.11	2.11 ± 0.11	83.51 (4)	< 0.001
*L*. *angustifolia*	0.48	0.28–1.03	1.45 ± 0.81	0.46 ± 0.57	34.52 (4)	< 0.001

LC_50_, concentration of EO that kills 50% of the eggs. Data are calculated by Probit regression analysis and given as μL insect^-1^; CI, confidence Interval; df, degrees of freedom; *P*, significance level of Pearson Goodness-of-Fit Test.

**Fig 2:**
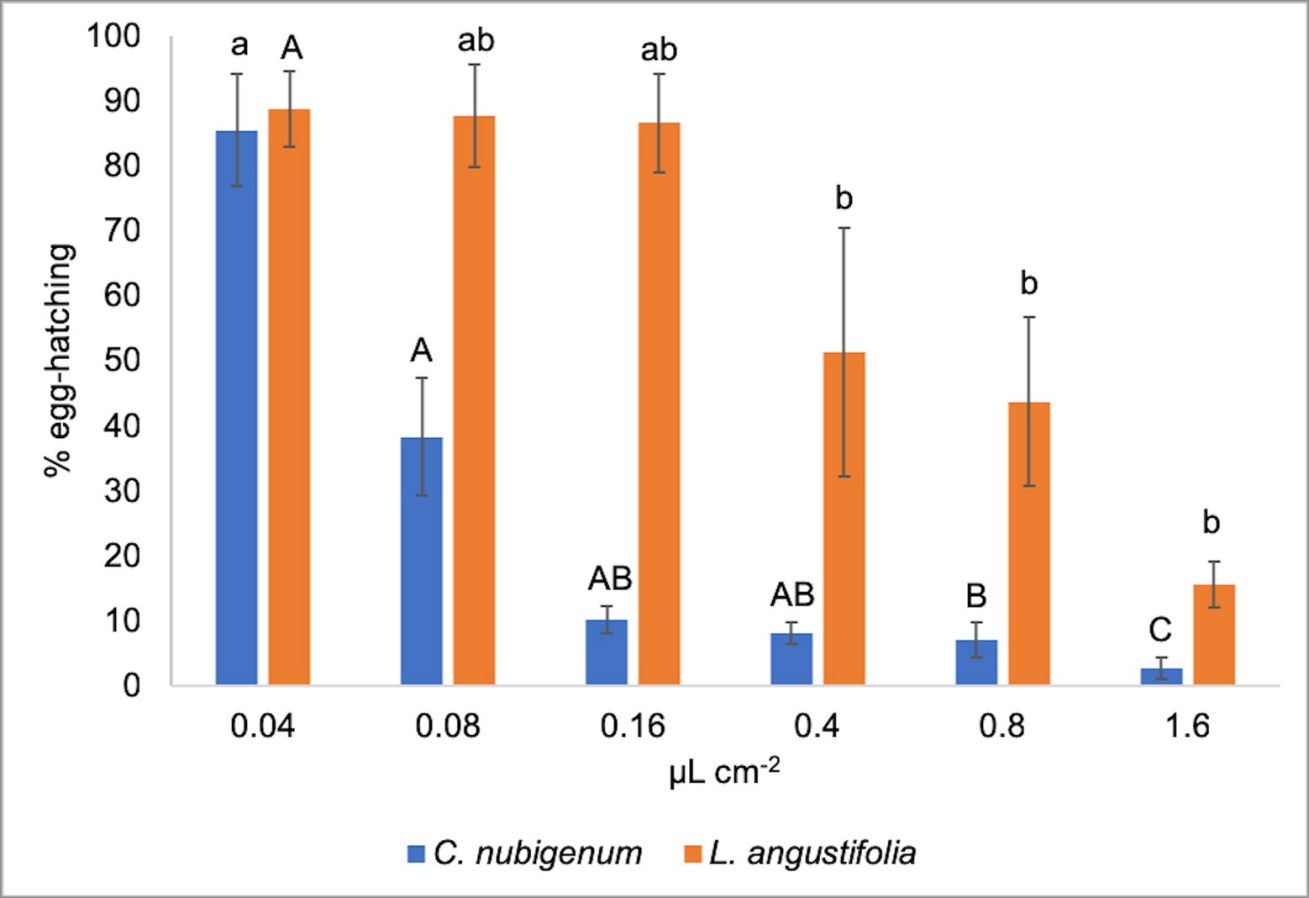
Toxicity of *Clinopodium nubigenum* and *Lavandula angustifolia* essential oils (EOs) against *Lucilia sericata* eggs. Histograms represent the mean percentage of egg-hatching after 24h of exposition to the EOs. Blue columns, *C*. *nubigenum* EO; orange columns, *L*. *angustifolia* EO. Bars represent standard errors. Different letters indicate significant differences among means (Tukey HSD, *P* ≤ 0.05). Capital letters indicate differences among *C*. *nubigenum* EO concentration; lowercase letters indicate differences among *L*. *angustifolia* EO concentration.

The RMP analysis showed that such differences in toxicity were significant (*L*. *angustifolia vs C*. *nubigenum* RMP = 6.899 (2.635–36.081)). More specifically, the *C*. *nubigenum* EO did reduce the egg-hatching up to 97.3% with a dose of 1.6 μL cm^-2^, with no significant differences among the concentrations from 0.16 to 1.6 μL cm^-2^, while the maximum reduction in the hatching of the eggs treated with *L*. *angustifolia* EO was 84.3% with no significant differences among concentrations, from 0.4 to 1.6 μL cm^-2^ (Fig 2).

### Adulticidal activity

The two EOs showed to have a clear adulticidal activity, by topical application, against the fly *L*. *sericata* even at low doses. More specifically, the LD_50_ values of the EOs were 0.28 to 0.39 μL per individual for *C*. *nubigenum* and *L*. *angustifolia*, respectively (Table 4). Relative toxicity, calculated by RMP analysis, showed that the EO of *C*. *nubigenum* was significantly more effective than the *L*. *angustifolia* one (*L*. *angustifolia vs C*. *nubigenum* RMP = 1.417 (1.125–1.836)).

**Table 4:** Toxicity of *Clinopodium nubigenum* and *Lavandula angustifolia* essential oils (EOs) against adults of *Lucilia sericata*.

EO	LD_50_	95% CI	Slope ± SE	Intercept ± SE	χ2 (df)	*P*
*C*. *nubigenum*	0.39	0.35–0.46	2.77 ± 0.30	1.12 ± 0.17	2.40 (2)	0.301
*L*. *angustifolia*	0.28	0.23–0.33	2.69 ± 0.30	1.50 ± 0.19	2.87 (2)	0.238

LD_50_, dose of EO that kills 50% of the insects. Data are calculated by Probit regression analysis and given as μL insect; CI, Confidence Interval; df, degrees of freedom; *P*, significance level of Pearson Goodness-of-Fit Test.

### AChE inhibition

**Fig 3:**
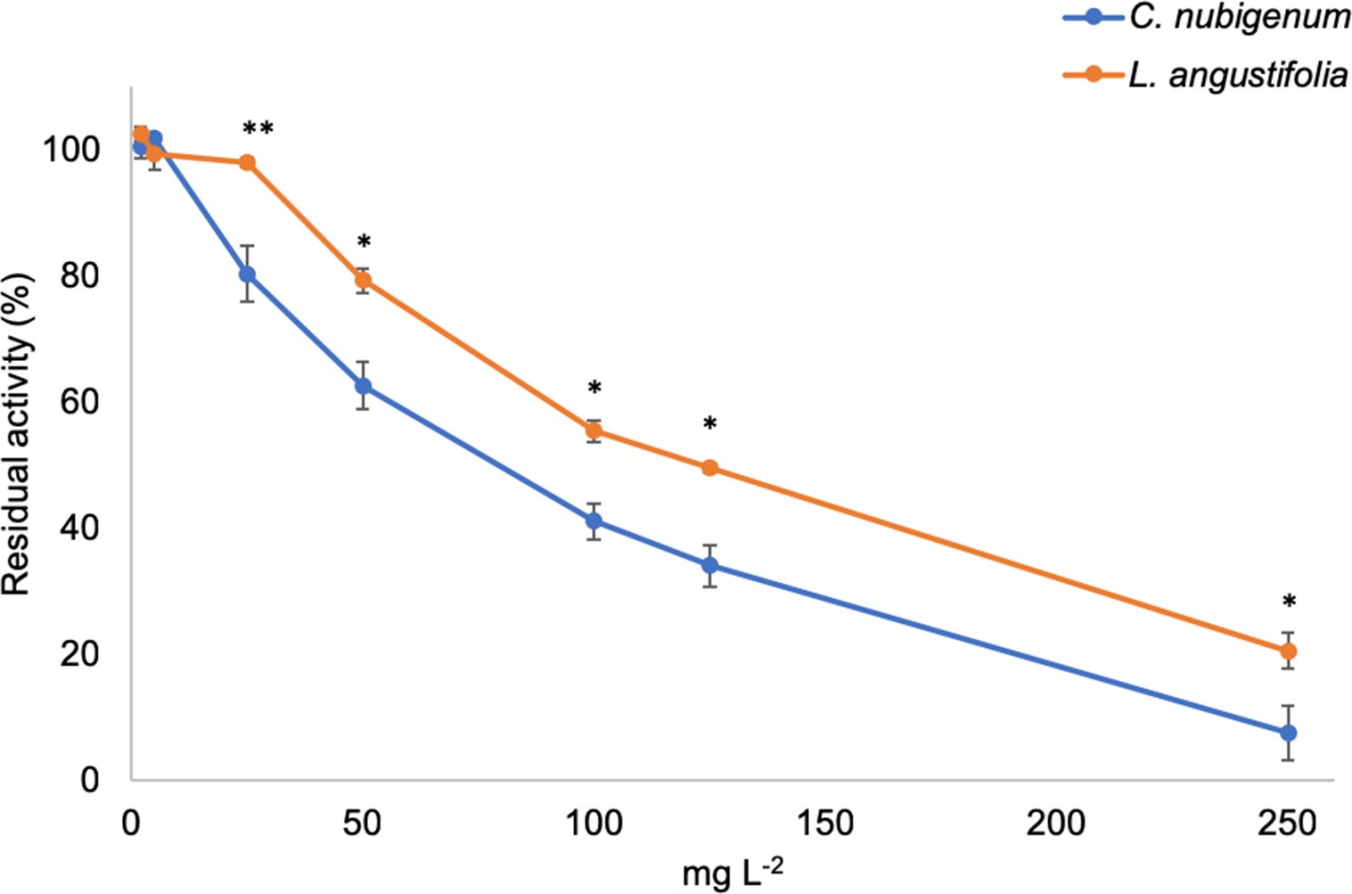
Acetylcholine esterase (AChE) inhibition by *Clinopodium nubigenum* and *Lavandula angustifolia* essential oils (EOs). Data are expressed as percentage of the AChE residual activity and represent the mean of three replicates. Bars represent standard errors. Asterisks represent a significant difference between the EOs (Student’s t test, *P* ≤ 0.05).

The AChE inhibitory activity of the two EOs is shown in Fig 3. The ANOVA showed significant differences between the inhibitory activity of the two EOs (*F*_1, 28_ = 60.140; *P* < 0.001), with a significant effect of the dose (*F*_6, 28_ = 315.589; *P* < 0.001) and the interaction oil *x* dosage (*F*_6, 28_ = 4.512; *P* = 0.003).

Generally speaking, the EO of *C*. *nubigenum* was found to be a stronger *in vitro* inhibitor of *L*. *sericata* AChE (IC_50_ = 67.450 mg L^-1^; R^2^ = 0.909; d.f. = 20) than the EO of *L*. *angustifolia* (IC_50_ = 79.495 mg L^-1^; R^2^ = 0.907; d.f. = 21).

### Antimicrobial activity

Both the EOs showed to have significant antibacterial activity, the intensity of which varied depending on the microbial strain (*F*_4, 139_ = 11.745; *P* < 0.001) and the EO concentration (*F*_1, 139_ = 239.925; *P* < 0.001) but not on the EO (*F*_1, 139_ = 0.320; *P* = 0.857) and with no significant interaction between the EO and the microbial strain (*F*_4, 139_ *=* 1.591; *P* = 0.180).

The microbial growth inhibiting effect of the EOs as measured by the agar disc diffusion method is presented in Table 5. At the highest dose (10 μL disc^-1^), the zone of inhibition of the EO of *C*. *nubigenum* ranged from 14.7 ± 0.7 to 45.0 ± 0.0 mm for *S*. *abaetetuba* and *C*. *albicans*, respectively. Similarly, the microbial growth inhibiting effect of the EO of *L*. *angustifolia* ranged from 14.7 ± 0.3 and 45.3 ± 1.7 mm for *S*. *abaetetuba* and *E*. *coli*, respectively. A post-hoc pairwise comparison showed that *S*. *abaetetuba* was the most resistant strain (Bonferroni, *P* ≤ 0.001). Consistently, the MIC and MLC values showed that the most generally susceptible microbial pathogen was the *C*. *albicans* with MIC and MLC values of *C*. *nubigenum* EO of 0.63 and 2.5 μL mL^-1^, respectively and MIC and MLC values of *L*. *angustifolia* EO of 1.25 and 5 μL mL^-1^, respectively (Table 6). The most resistant microbial strain was *S*. *abaetetuba* with values of 10 μL mL^-1^ or above for both the EOs (Table 6).

**Table 5:** Antimicrobial activity (zone of inhibition, mm) of *Clinopodium nubigenum* and *Lavandula angustifolia* essential oils (EOs) against *Escherichia coli*, *Bacillus subtilis*, *Streptococcus aureus*, *Candida albicans*, and *Salmonella abaetetuba* strains.

EO	Dose^a^	*E*. *coli*	*B*. *subtilis*	*S*. *aureus*	*C*. *albicans*	*S*. *abaetetuba*
*C*. *nubigenum*	10	20.9 ± 0.5	33.0 ± 2.0	36.0 ± 4.9	45.0 ± 0.0	14.7 ± 0.7
	5	19.7 ± 0.3	18.0 ± 1.5	18.7 ± 1.3	33.3 ± 9.3	13.0 ± 1.5
	2.5	18.0 ± 2.1	15.7 ± 0.7	15.3 ± 0.3	17.0 ± 0.6	13.0 ± 0.6
	1.25	18.0 ± 0	14.7 ± 1.3	14.3 ± 0.7	15.0 ± 1.5	12.7 ± 0.9
	0.63	18.0 ± 0.6	13.7 ± 0.9	13.7 ± 0.3	15.3 ± 0.3	10.3 ± 4.7
*L*. *angustifolia*	10	45.3 ± 1.7	42.7 ± 2.3	45.0 ± 0.0	43.3 ± 1.7	14.7 ± 0.3
	5	19.7 ± 0.9	20.3 ± 1.5	17.3 ± 1.3	17.3 ± 1.3	15.0 ± 1.0
	2.5	16.7 ± 1.9	15.0 ± 0.6	15.7 ± 0.7	16.3 ± 0.7	13.3 ± 0.9
	1.25	15.3 ± 0.7	10.0 ± 0.6	11.7 ± 0.9	13.7 ± 0.9	13.0 ± 0.6
	0.63	15.7 ± 0.9	10.7 ± 0.3	9.7 ± 0.7	14.33± 0.9	9.7 ± 0.9

^a^, μl disc^-1^.

Data represent the diameter (mm) of the zones of inhibition of the EOs as measured by the agar disc diffusion test (mean ± standard error).

**Table 6:** Minimum inhibitory concentration (MIC) and minimum lethal concentration (MLC) values of the essential oils of *Clinopodium nubigenum* and *Lavandula angustifolia* against *Escherichia coli*, *Bacillus subtilis*, *Streptococcus aureus*, *Candida albicans*, and *Salmonella abaetetuba* microbial strains.

Pathogen	*C*. *nubigenum*		*L*. *angustifolia*	
	MIC	MLC	MIC	MLC
*E*. *coli*	1.25^a^	2.50	0.63	> 10.00
*B*. *subtilis*	5.00	> 10.00	1.25	> 10.00
*S*. *aureus*	2.50	10.00	2.50	> 10.00
*C*. *albicans*	0.63	2.50	1.25	5.00
*S*. *abaetetuba*	> 10.00	> 10.00	10.00	> 10.00

Values are given as μl ml^-1^

## Discussion

Health safety and environmental concerns about synthetic pesticides have led to restrict their use and look for safe alternatives to control insects and other pests. EOs, classed as botanical pesticides, have shown to have remarkable potential as effective eco-friendly biocides and successful insect pest repellents. To date, however, very few studies have been performed on their use against myiasis-inducing species of blowflies.

In this study, we tested the EOs extracted from *C*. *nubigenum* and *L*. *angustifolia*. While *L*. *angustifolia* is a well-known aromatic plant, the EO of which has been investigated in many trials in the past, only a few reports have been published about the composition of the EO of *C*. *nubigenum* [48; 49]. Monoterpenes, particularly the oxygenated ones, seem to be the main components in all the reported essential oil compositions of *C*. *nubigenum* specimens. Ruiz et al. [48] hydrodistilled samples of *C*. *nubigenum* from Ecuador and the EO was mainly rich in oxygenated monoterpenes, the most abundant of which was carvacryl acetate (38.1%). This was followed by carvacrol, accounting for 29.0%. Gilardoni et al. [49] also described the composition of the EO extracted from both dried and fresh plants from Ecuador and the specimens had a pulegone chemotype (37.11 and 72.79% in the dried and fresh samples, respectively). In the dried sample, it was followed by menthone with a relative abundance of 11.57%, whilst, only traces of it could be found in the fresh sample, where the second most abundant compound was linalool (7.81%).

Although different in their chemical composition, the EO of *C*. *nubigenum* and *L*. *angustifolia* EOs, showed to have a clear toxic activity against *L*. *sericata*. In this respect, the results showed that *C*. *nubigenum* and *L*. *angustifolia* are toxic by contact and/or fumigation against the eggs and adults of the fly and can inhibit its oviposition as well. To the best of our knowledge, this is the first report about the toxic and oviposition deterrent activity of *C*. *nubigenum* against insects. On the contrary, the effectiveness of the EO of *L*. *angustifolia* has been observed in several trials against Diptera [50; 51; 52], Lepidoptera [53] and Coleoptera species [54; 55]. In our experiment, we found the EO of *C*. *nubigenum* to be more toxic than that of *L*. *angustifolia*. This could be due to the different chemical composition of the two EOs. Because of such interspecific chemical variability, Bedini et al. [6] found a very different level of toxicity when comparing two EOs extracted from *Artemisia annua* and *A*. *dracunculus* against the blowfly *Calliphora vomitoria*.

In this trial, the EO of *C*. *nubigenum* was found to be more effective than that of *L*. *angustifolia* also in terms of oviposition deterrence. Even though both the EOs did almost completely deter oviposition for up to 3 h after the treatment, the EO of *C*. *nubigenum* was the most persistent one maintaining a good oviposition deterrence activity even at the lower dose after 24 hours after the treatment. In keeping with our results, a strong oviposition deterrence was also previously observed for the EOs of *A*. *annua* and *A*. *dracunculus* against the blowfly *Calliphora vomitoria* [6] and for *Lucilia cuprina*, in media treated with *Melaleuca alternifolia* EO [56]. In comparison, the effectiveness of the EO of *C*. *nubigenum* (OD% = 72.61 at 2% EO) seems to be in line with those of *A*. *annua* and *A*. *dracunculus* EOs (OD% = 69.31 and 96.77, respectively, at 2.5% EO). The different effectiveness of the two EOs indicates that the EOS oviposition deterrent effect may depend not only on the different chemical composition of the EOs but also on the target species. Moreover, the complexity of the insects’ sensory system makes it difficult to understand how the chemical information encoded in the deterrent molecules is perceived by the insect (through olfactory, gustatory or other receptors on the ovipositor or perhaps tarsi) and triggers its behavioural response.

To the best of our knowledge, no study concerning the AChE inhibitory activity of the EOs from *C*. *nubigenum* and *L*. *angustifolia* has been reported so far. Our results (IC_50_ = 67.450 and 79.495 mg L^-1^ for *L*. *sericata* and *L*. *angustifolia*, respectively) are in agreement with the AChE inhibitory activity of *Salvia lavandulaefolia* Vahl., *Eucalyptus camaldulensis* Dehnhardt and *Ocimum canum* Sims, with (IC_50_ of 50.0, 18.0 and 36.0 μg/mL, respectively) [57] while, the AChE inhibitory effect of *C*. *nubigenum* and *L*. *angustifolia* turned out to be stronger than the one observed for the *A*. *annua* and *A*. *dracunculus* EOs (IC_50_ of 202.6 and 472.4 μg/mL, respectively) [6]. This finding suggests that one of the effects of the EOs extracted from *C*. *nubigenum* and *L*. *angustifolia* against *L*. *sericata* is the inhibition of AChE activity. Therefore, the enzymatic test based on the inhibition of AChE could be a useful quick tool for further researches into the effectiveness of each single compound of both the EOs against *L*. *sericata*.

Flystrike flies usually lay their eggs near wounds or on moist, attractive areas of the sheep. Such preference for wounds and the ability of the larvae to abrade and penetrate in the tissues makes these flies also a source and carrier of microbial infections. EOs can prevent the spread of pathogens through their well-known antimicrobial activity. As expected, we found that *C*. *nubigenum* and *L*. *angustifolia* showed to have clear toxic, bacteriostatic and mycostatic activities against several pathogens. In particular, we found that the Gram-negative *S*. *abaetetuba* was the overall more resistant pathogen. To the best of our knowledge, there are no previous reports on the susceptibility of *S*. *abaetetuba* to essential oils of aromatic plant. However, Sahu [58], found that *S*. *abaetetuba* was susceptible to ethanolic extracts of four *Ocimum* species and a clear antimicrobial effect of EOs against *Salmonella* species were observed [59; 60]. On the contrary, we observed a high susceptibility of the pathogenic fungus *C*. *albicans* to essential oils. This is consistent with previous trials that found anti-candida properties in plants used in the Brazilian traditional medicine [61] and in EOs extracted from other aromatic plants such as *Myrtus communis* [62], *Mentha piperita* [63], *Origanum* spp. EOs [64; 65], *Artemisia annua* and *A*. *dracunculus* EOs [6].

Extensive evidence suggests that the observed antimicrobial effect of the EOs is due to their interaction with the cytoplasmic membrane of microorganisms. The hydrophobicity of the EOs [66] enables their chemical components to accumulate in cell membranes, interfering with their structures and increasing their permeability [67; 68; 69; 70]. The leakage of intracellular constituents and the impairment of the microbial enzymatic system can then lead the cell to death [71; 72; 73]. However, antimicrobial activity of the EOs, as well as their toxic and oviposition deterrent activity against insects cannot be attributed to one particular or specific mechanism [74]. In fact, such toxic effects (and most likely their toxicity to insects) can be due to a large number of different chemical components of the EOs, the synergistic or antagonistic effects of which do not always let the actual biological effect correlate with the type and quantity of the main components of the EOs [75; 76].

## Conclusions

The prevention of parasitic infections is a priority in animal husbandry. Using the EOs of *C*. *nubigenum* and *L*. *angustifolia* as protectant against the myiasis-inducing blowfly *L*. *sericata* may broaden the very narrow spectrum of eco-and animal welfare-friendly alternative options to synthetic pesticides and surgical procedures to control flystrike.

Besides, the exploitation of indigenous aromatic plants such as *C*. *nubigenum* EO may be a valuable additional resource for the economy of rural Andean communities. However, further studies are needed to test the actual applications of our laboratory results and to establish the right doses and the best methods to formulate and deliver such EOs, in the attempt to extend their effectiveness and minimize the number of treatments.

## Supporting information

S1 Appendix Excel spreadsheet containing raw data from the study. Each sheet contains the individual data obtained in a particular trial, as noted.
(XLSX)
